# Interaction of hallucinogenic rapid-acting antidepressants with mGlu2/3 receptor ligands as a window for more effective therapies

**DOI:** 10.1007/s43440-023-00547-4

**Published:** 2023-11-07

**Authors:** Barbara Chruścicka-Smaga, Agata Machaczka, Bernadeta Szewczyk, Andrzej Pilc

**Affiliations:** Department of Neurobiology, Maj Institute of Pharmacology Polish Academy of Sciences, Kraków, Poland

**Keywords:** Depression, Co-treatment, Glutamatergic system, Hallucinogens, Psychedelics, Rapid-acting antidepressants, mGlu2/3 receptors

## Abstract

The desire to find a gold-standard therapy for depression is still ongoing. Developing one universal and effective pharmacotherapy remains troublesome due to the high complexity and variety of symptoms. Over the last decades, the understanding of the mechanism of pathophysiology of depression and its key consequences for brain functioning have undergone significant changes, referring to the monoaminergic theory of the disease. After the breakthrough discovery of ketamine, research began to focus on the modulation of glutamatergic transmission as a new pharmacological target. Glutamate is a crucial player in mechanisms of a novel class of antidepressants, including hallucinogens such as ketamine. The role of glutamatergic transmission is also suggested in the antidepressant (AD) action of scopolamine and psilocybin. Despite fast, robust, and sustained AD action hallucinogens belonging to a group of rapid-acting antidepressants (RAA) exert significant undesired effects, which hamper their use in the clinic. Thus, the synergistic action of more than one substance in lower doses instead of monotherapy may alleviate the likelihood of adverse effects while improving therapeutic outcomes. In this review, we explore AD-like behavioral, synaptic, and molecular action of RAAs such as ketamine, scopolamine, and psilocybin, in combination with mGlu2/3 receptor antagonists.

## Introduction

Brain disorders represent one of the major threats to our society. Among them, depression is the most burdensome disorder of all diseases and the most costly of all brain diseases [1]. During the COVID-19 pandemic the number of people suffering from depression increased fourfold. As a result, depression has become the leading cause of disability affecting more than 300 million in their lifetime worldwide [2]. The classical antidepressant (AD) drugs which were introduced into the clinic over 70 years ago and affect the monoaminergic system are characterized by a slow onset of action, adverse effects, and a noticeable resistance rate [3]. Since effective pharmacotherapy of depression without significant adverse effects is still not available there is a large need for novel treatment strategies with faster onsets of action, higher remission rates, better efficacy in resistant cases, greater anti-suicide properties, and significant reduction of adverse effects.

## Co-administration as a promising approach for more effective AD pharmacotherapy

The co-administration approach has been successfully explored in preclinical studies [4] and clinical trials [5]. Targeting two different mechanisms instead of one is more relevant concerning the great complexity of psychiatric disorders. Accumulating evidence has shown that using two substances instead of searching for an all-in-one has more potential and can cover more disease symptoms if chosen wisely. Additionally, it allows for the use of lower doses of both substances, which is beneficial regarding the potential significant reduction of side effects [6]. Selective co-treatment allows the maintenance of an ideal balance between antidepressant outcomes and side effects. In this review, we would like to focus on describing AD's effects of simultaneous co-administration of mGlu2/3 receptor ligands with hallucinogens belonging to a group of rapid-acting antidepressants (RAA).

## Glutamatergic neurotransmission and depression

The glutamatergic system is the main excitatory neurotransmitter system in the central nervous system (CNS), and glutamatergic neurons are the most numerous and most widespread group of neurons. Over half of the 100 billion neurons in the CNS release glutamate (Glu) into the synaptic cleft and almost all neurons express receptors that are sensitive to Glu [7]. Therefore it’s not surprising that dysregulation of glutamatergic transmission throughout the brain is of great importance in the pathophysiology of several mental diseases, including depression. Glu, the main excitatory neurotransmitter in the brain acts via stimulation of the two major groups of receptors; ionotropic glutamate (iGlu) receptors and metabotropic glutamate (mGlu) receptors diversified in structure and pharmacological profile [8]. iGlu receptors are responsible for fast synaptic transmission (milliseconds) and include α-amino-3-hydroxy-5-methyl-4-isoxazolepropionic acid (AMPA), *N*-methyl-d-aspartate (NMDA), and kainite (KA) subtypes. Metabotropic receptors belong to the C class of GPCRs and by activating secondary messenger pathways possess modulatory effects on cell response. 8 subtypes of mGlu receptors have been distinguished and classified into three groups (group I consists of mGlu1 and 5, group II consists of mGlu2 and 3, and group III consists of mGlu4, 7, and 8 subtypes) based on similarity in the amino acids sequence, type of signaling pathway, pharmacological profile, and the location at the level of synapses and brain structures.

Over the past 30 years accumulating evidence has generated considerable interest in the field to target glutamate neurotransmission for the development of a novel class of antidepressants. Glutamatergic transmission attracted attention since pioneering studies of Trullas and Skolnick demonstrating the antidepressant (AD) action of NMDA receptor antagonists which contributed to the NMDA/glutamate theory of depression [9, 10]. Subsequent studies explored the AD-like properties of several NMDA receptor antagonists as well as AMPA receptor potentiators with varying degrees of success [11–13]. The first papers demonstrating the AD-like activity of mGlu receptor ligands including mGlu5 receptor antagonist [14] and mGlu7 receptor potentiator [15] came from our laboratory in Kraków and were further explored by others [16, 17]. The AD-like properties of group II antagonist were shown for the first time by the Japanese group of dr Chaki [18]. Although none of the AMPA, mGlu5 or mGlu7 receptor ligands have so far demonstrated significant AD efficacy in clinical trials, modulation of mGlu2/3 receptors continues to hold promise for the development of safer and more efficacious antidepressants [19].

## mGlu2/3 receptor antagonists

**Fig. 1:**
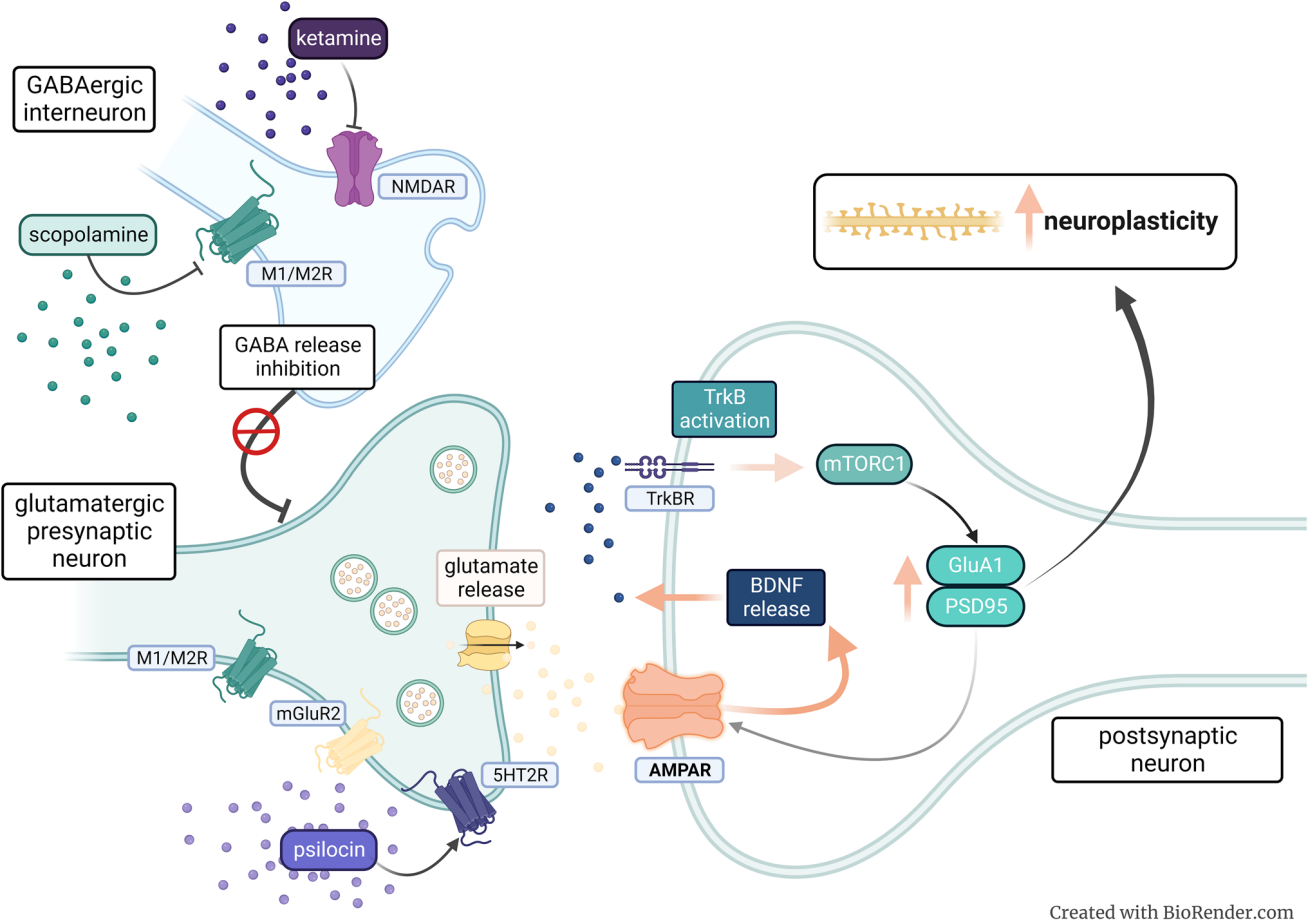
Postulated mechanisms of rapid-acting antidepressants action. The efficacy of RAA especially ketamine is related to glutamate, which is an initial and essential player in the mechanism of AD action. According to the disinhibition hypothesis ketamine blocks the NMDA receptors on the GABAergic interneurons innervating glutamate pyramidal cells. Scopolamine blocks muscarinic receptors type 1 and/or 2 on the same neurons. Both mechanisms leads to the blockage of GABAergic neurotransmission. In consequence, activation of pyramidal cells innervated by GABA interneurons leads to the release of glutamate into the synaptic cleft. The glutamate burst fosters enhanced functioning of AMPA receptors (an augmenting the potency of AMPA receptors), causing the release of BDNF, activation of TrkB receptor, and stimulation of the AKT/ERK/mTOR pathway. This sequence of events results in the local synthesis of synaptic proteins (PSD95, GluA1) which is accompanied by dendritic spine growth and improved connectivity in the brain. Psilocin, the active metabolite of psilocybin exerts similar behavioral and cellular AD-like effect. This may suggest overlapping mechanisms initiated by the modulation of serotonin receptors, predominantly the postsynaptic 5-HT2 receptors, which may affect a glutamate-dependent increase in the activity of pyramidal neurons in the PFC

mGlu2 and mGlu3 receptors belong to group II mGlu receptors which are coupled to Gi/o proteins. Activation of mGlu2/3 receptors inhibits adenylate cyclase and decreases the level of second messenger cAMP [20]. They are mainly localized in the cortical and limbic areas of the brain, predominantly presynaptically where they function as autoreceptors or heteroreceptors to negatively regulate the release of Glu or GABA [21–23]. A number of studies showed that mGlu2/3 receptor antagonists produce rapid and prolonged AD-like properties in rodents which qualifies them to the group of rapid-acting antidepressants (RAA) [4, 18, 24], for review see [25]. Interestingly, the mechanism of AD-like actions of mGlu2/3 receptor ligands appears to be similar to that of other RAA including the NMDA receptor antagonist, ketamine (Fig. 1). Briefly, the blockade of presynaptic mGlu2/3 receptors leads to increased glutamate release, stimulation of postsynaptic AMPA receptors, increased release of BDNF (brain-derived neurotrophic factor), and activation of the mammalian target of rapamycin (mTOR) pathway. All together leads to an increase in spine formation which is considered a mechanism for long-lasting AD effects [26, 27]. In addition, mGlu2/3 receptor antagonists have been shown to produce fewer adverse effects than ketamine [28]. Taken together this may suggest potential similar AD efficacy with safer adverse effects profile in humans.

The clinical studies conducted so far did not show the AD outcome of mGlu2/3 receptor negative allosteric modulator (NAM), decoglurant [29]. Decoglurant was well tolerated overall but did not exert any AD activity in patients with partially refractory major depressive disorder [29]. A high placebo response and the lack of defined target engagement might be the reason for these negative results [27] (see [30] for discussion). Currently, clinical trials with the use of mGlu2/3 receptor blockers such as TS-161 (orthosteric antagonists) and DSP-3456 (NAM) are underway [30]. This still gives hope for the development of safer and more efficacious antidepressants based on mGlu2/3 receptors modulation.

## Hallucinogens as rapidly acting antidepressants (RAA)

Hallucinogens are described as psychoactive drugs producing altered states of consciousness. It is a very large and diverse group of substances that can be divided into three major classes: dissociatives, deliriants, and psychedelics. Within each group RAA such as ketamine, scopolamine, and psilocybin with overlapping mechanisms of action can be found [31].

## The RAA—Ketamine

Ketamine is a noncompetitive antagonist of NMDA receptors. Ketamine has long been used as an anesthetic agent with its dissociative adverse effects and abuse potential. Edward Domino was the first who used ketamine for general anesthesia in 1964 [32]. In the review paper entitled ‘Taming the Ketamine Tiger’ he described a patient who was trying to tell him that ketamine is a much better antidepressant than traditional ones. Since he was aware of the psychotomimetic effects of ketamine he discouraged the patient from taking the drug [32]. If he had listened to the patient, the AD effects of a subanesthetic dose of ketamine could have been discovered years before 2000 when they were described for the first time [33]. These results demonstrated for the first time that a rapid and long-lasting AD effect is achievable. The effectiveness of ketamine has been further confirmed in treatment-resistant depression (TRD), where AD effects of ketamine appeared 110 min after administrating a single subanesthetic dose, and its effect lasted 7 days [34]. A recent meta-analysis of randomized clinical trials confirmed the rapid and robust AD effects of a single dose of ketamine in unipolar and also in bipolar depression, and that repeated administration of ketamine effectively maintained the initial AD effects of a single dose [35].

Ketamine has been shown to be effective also in several animal models of depression which gave a great opportunity to study the mechanism of its AD action (Fig. 1) [36]. One of the most empirically supported is the disinhibition hypothesis which postulates that ketamine blocks excitatory NMDA receptors located on GABAergic interneurons causing increased glutamate release from pyramidal neurons [37]. This mechanism initiates series of other molecular and cellular events that contribute to the AD outcome of ketamine. Briefly, glutamate released from the presynaptic terminals binds and activates postsynaptic AMPA receptors [38]. Activation of AMPA receptors leads to the release of BDNF, phosphorylation of tropomyosin receptor kinase B (TrkB), stimulation of the mTOR pathway, and in consequence increased synaptogenesis [39, 40]. Since glutamate disinhibition mechanism, doesn’t explain why other NMDA receptor antagonists don’t show such RAA activity as ketamine, alternative hypothesis are still in development (for review see [41, 42]).

Ketamine is a mixture of two racemic forms (*S*)-ketamine and (*R*)-ketamine [43]. Both isoforms have been shown to produce AD-like effects in animal models of depression, while AD effects in human are more pronounced for (S)-ketamine [44, 45]. The intranasal esketamine in conjunction with an oral antidepressant was recently approved for use in adults with TRD [46]. Despite a great revolution that ketamine brought as a “new age antidepressant agent” it is also a drug of abuse with several adverse effects such as cognitive and motor impairments, transient and moderate dissociative reactions, as well as psychotomimetic effects. Hence the treatment strategies allowing to reduce the dose of ketamine would be beneficial to the patient making them highly desirable.

Interestingly, co-administration of ketamine with mGlu2/3 receptor antagonist LY341495 allowed for around tenfold decrease in dosage of both substances in animal model of depression. Both rapid and sustained AD-like activity has been demonstrated, along with a significant reduction of adverse effects typical of ketamine including short-term memory impairments, hyperlocomotion, and motor coordination [4, 47]. Such a combination has been also shown to increase activation of mTOR pathway and synaptic protein level suggesting that the observed AD-like action is dependent on AMPA receptors stimulation and TrkB receptors signaling [4]. Further studies showed that ketamine metabolite (2R, 6R)-HNK also produces AD-like effects in rodents and these effects are independent of NMDA receptors inhibition [48]. Recently, the AD-like action of subeffective doses of mGlu2/3 antagonist LY341495 with (2R, 6R)-HNK have been also demonstrated in rodents [49]. These results strongly support the use of ketamine and its metabolite in combination with mGlu2 receptors antagonists in clinical trials for TRD.

## The RAA—Scopolamine

The cholinergic hypothesis of depression was proposed in the early 1970s based on the observations that acetylcholinesterase inhibitor, physostigmine exaggerated depressed mood in patients with both unipolar and bipolar depression [50, 51]. However, it took over 30 years till rapid, robust and sustained antidepressant effects of the antimuscarinic drug scopolamine have been described [52]*.* Scopolamine (another name Hyoscine) is a naturally derived alkaloid of species from the Solanaceae family. It is widely used as a butyl bromide salt that does not penetrate into the brain in the treatment of abdominal pain, irritable bowel syndrome, and bladder spasms. Hydrobromide derivative of scopolamine that enters the brain is mainly known as a motion sickness reliever and a preoperative medication. Scopolamine is a nonselective antagonist of all five muscarinic receptor subtypes (M1–M5) [53]. Despite the exact mechanism of AD action of scopolamine is not known it has been suggested that blockage of M1 and M2 receptors on GABAergic interneurons may be involved (Fig. 1) [53, 54]. Scopolamine is also known to cause significant adverse effects including memory impairment [55], drowsiness, as well as visual disturbances [56], which hamper its use as a psychiatric drug. Moreover, recent clinical data indicated that scopolamine exerts AD effects of varying intensity [57]. Therefore, there is a need for more research to establish its potential therapeutic efficacy and safety profile.

The mechanism of AD action of scopolamine overlaps with the AD effects of RAA including ketamine, psychedelics and mGlu2/3 receptor blockers in terms of AMPA receptor activation and modulation of serotonergic neurotransmission. Results from our group have demonstrated enhancement of the therapeutic effects of scopolamine following co-administration with a mGlu2/3 receptor antagonist, LY341495. A profound fast AD-like effects were seen in the tail suspension test and in the forced swim test in mice. In addition, the observed therapeutic effects have been shown to be dependent on AMPA receptors modulation and independent of serotonergic system activation. What’s more, combined administration of subeffective doses of both substances allowed for a significant reduction of adverse effect driven by scopolamine [58]. Although more studies are needed to confirm the above effects, joint administration of low doses of scopolamine and LY341495 might be an effective and potentially safer strategy in the therapy of depression.

## The RAA—Psychedelics

The AD effects of psychedelics known as classical serotonergic hallucinogens were described about 10 years before the discovery of antidepressants acting on monoaminergic systems [59]. The serotonergic hallucinogens are one of the most potent agonists of 5-HTR2A, the main excitatory G-coupled protein receptor (GPCRs) of all 5-HT receptors, primarily expressed in the frontal cortex [60]. Serotonergic psychedelics are known to exert rapid AD effects in humans [61, 62] and AD-like effects in rodents [63, 64]. Given the potential rapid therapeutic efficacy of psychedelics, there is renewed interest in the use of these compounds in the context of major and resistant depression [65]. It seems that among all psychedelics showing AD activity, psilocybin (mainly its active metabolite, psilocin) is characterized by the lowest physiological toxicity and abuse liability having the safest profile. Its use, however, is not free of adverse effects like overwhelming distress during drug action (“bad trip”) or persisting perceptual disturbances [66, 67]. New wave of placebo-controlled, randomized trials showed promising therapeutic outcomes and no serious adverse effects. Most side effects including anxiety, confusion, headache, and nausea were mild to moderate and transient. However, there is no evidence for long-term safety and efficacy following more than one or two doses. In addition, AD effects need to be confirmed in a much bigger group of patients. Therefore, more research is needed to establish the therapeutic safety profile of psychedelics.

It’s well established that modulation of mGlu2/3 receptors plays an important role in the behavioral and cellular effects of hallucinogenic drugs, including 5-HTR2A agonists. mGlu2/3 receptor antagonists have been shown to increase an extracellular serotonin concentration in the medial prefrontal cortex (mPFC) and the firing rate of serotonin neurons in the dorsal raphe nucleus [68]. The growing evidence emphasizes the role of the glutamate system in the 5-HTR2A-mediated effects on brain function. Specifically, it has been suggested that activation of 5-HTR2A leads to a glutamate-dependent increase in the activity of pyramidal neurons in the PFC modulating network activity [69]. Recent data showed overlapping action of psilocin and mGlu2/3 receptor antagonists on structural and functional changes in synaptic strength in PFC, effects strongly related to AD efficacy [70]. On the other hand, mGlu2/3 receptor antagonist, LY341495 has been shown to significantly decrease the hallucinogenic-like effects of LSD driven by 5-HT2A receptors in the head-twitch behavior of mice [71]. The exact mechanism underlying the AD action of psychedelics is not known. It’s widely thought that modulation of 5-HT2A receptors and altered consciousness is required for their therapeutic effects (Fig. 1) [64]. However, more recent studies demonstrated that pretreatment with 5-HT2A/2C receptor antagonist, ketanserin didn’t block AD-like effects (both cellular and behavioral) of psilocybin [65]. These results may suggest other potential mechanisms to be involved in the observed rapid and/or sustained AD outcomes of psychedelics.

Considering the above, the combination of subthreshold doses of psychedelics with mGlu2/3 receptor antagonists would be interesting from the point of view of potentially reducing adverse effects and developing a safer therapeutic strategy. Additionally, it may also provide insights into the mechanisms of action of psychedelics, specifically whether an alteration of consciousness is necessary for their robust therapeutic effects.

## Potential molecular mechanisms of the synergistic AD action of hallucinogens and mGlu2/3 receptors ligands

**Fig. 2:**
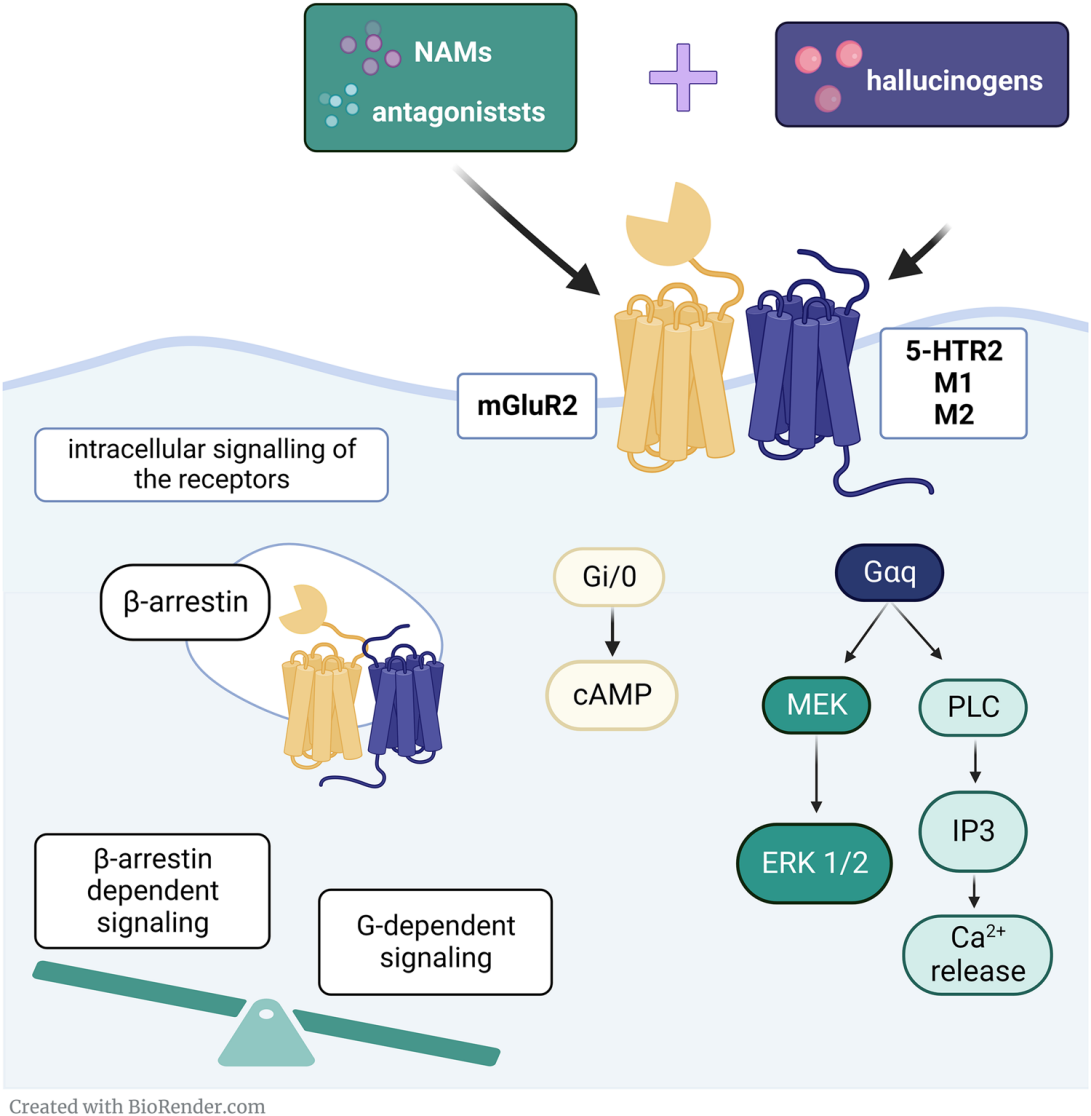
Potential molecular mechanisms of simultaneous co-administration of hallucinogens with mGluR2/3 ligands. The G-protein-mediated intracellular signaling pathways activated by hallucinogens lead to the activation of further downstream messengers, contributing to changes in the expression of molecules involved in neuroplasticity a fingerprint of AD outcome. β-arrestin binding to the receptor leads to its desensitization and internalization, a well-known mechanism of protecting the cell from overstimulation. Activation of 5-HT2 receptors or antagonism of M1-2 receptors with simultaneous blockage of mGlu2/3 receptors may shift G-dependent and β-arrestin mediated signaling balance and change significantly therapeutic outcomes of RAAs. One of the potential mechanisms for this phenomenon is the formation of functional heterocomplexes of target receptors, a phenomenon that gives another level of complexity in signaling modulation

The mechanisms of how both activation by psychedelic and non-psychedelic agonists and blockade by traditional antidepressants or more selective antagonists of the target receptors including NMDA, mGluR2/3, M1, M2, and 5-HT2 receptors, can produce AD-like activity in animal models of depression is still an open question. One of the potential explanations is the location of the target receptors at the level of brain structure and type of cell as in the case of potential AD mechanisms of ketamine. Second, is the involvement of other off-target receptors as well as the concept of biased agonism in which binding to the same receptor modulates different signaling pathways. The best example here is psilocybin which possesses affinity to most of serotonin receptors and its AD action is postulated to be driven by 5-HTR2A dependent β-arrestin mediated signaling [72]. Another potential mechanism is the specific modulation of downstream signaling driven by the formation of functional receptor heterooligomers (heterocomplexes) (Fig. 2).

GPCRs function as homo- (receptors from the same group) and heterooligomers (receptors from different groups). Oligomerization is now considered to be a fundamental process for the proper functioning of these receptors. Heterooligomers of specific GPCRs are characterized by unique biochemical, functional, and pharmacological properties compared to their homooligomeric counterparts, creating an additional level of regulation of signal transduction in the brain. Disturbances in the formation and functioning of GPCR heterooligomers are associated with an increased risk of mental disorders [73]. For example, disruption in the formation of mGlu2/5-HT2A receptor heterocomplexes in distinct brain regions as well as changes in their signaling have been proposed in psychosis [74]. This strongly suggests that the modulation of psychomimetic effects of hallucinogenic drugs may be partially driven by the signaling of this heterocomplex [75]. Therefore, mGlu2/5-HT2A receptor heterocomplex mediated signaling cannot be ignored in the context of synergistic modulation of these two main neurotransmitter systems in the pathophysiology of depression and AD action of halucinogens. Until today only a few articles have been published on the possible role of distinct heterocomplexes in the pathophysiology of depression [76]. Demonstrating that modulation of specific heterocomplex mediated signaling following co-treatment with hallucinogenic drugs and mGluR2/3 antagonists may work as a molecular switch between psychosis and AD activity [64], may fill the gap in knowledge about complex processes in the brain and open new avenues towards developing more effective new-age therapies for depression.

## Concluding remarks

A better understanding of the nature of interactions between mGlu2/3 receptors and hallucinogenic substances can provide potential molecular mechanisms related to complex processes in the brain and thus contribute to the development of safer, more effective, so-called new-age pharmacotherapies for depression. The creation of complex AD therapies must be carefully examined in terms of the metabolism pathways of the proposed drugs, their potential interactions in the nervous system and other tissues, and both short and long-term effects. Therapies based on co-treatment allow for a more comprehensive approach through different drug action points, thereby increasing the spectrum of symptoms that the drug can act on while maintaining a safer profile by using lower doses of the substances. This approach makes it possible to construct a more tailored therapy to the spectrum of the individual patient's symptoms.

## Data Availability

Data sharing is not applicable to this article as no datasets were generated or analysed during the current study.

## Abbreviations

AD: Antidepressant
RAA: Rapid-acting antidepressant
CNS: Central nervous system
Glu: Glutamate
iGluR: Ionotropic glutamate receptors
mGluR: Metabotropic glutamate receptors
mGlu2/3: Metabotropic glutamate receptors type 2/3
AMPA: α-Amino-3-hydroxy-5-methyl-4-isoxazolepropionic acid receptors
NMDA: *N*-Methyl-d-aspartate receptors
M1-5: Muscarinic receptors type 1–5
5HT2A: Serotonin receptors type 2A
BDNF: Brain-derived neurotrophic factor
mTOR: Mammalian target of rapamycin pathway
NAM: Negative allosteric modulator
GPCRs: G-coupled protein receptors
